# Genetic variants associated with two major bovine milk fatty acids offer opportunities to breed for altered milk fat composition

**DOI:** 10.1186/s12711-022-00731-9

**Published:** 2022-05-26

**Authors:** Tim Martin Knutsen, Hanne Gro Olsen, Isaya Appelesy Ketto, Kristil Kindem Sundsaasen, Achim Kohler, Valeria Tafintseva, Morten Svendsen, Matthew Peter Kent, Sigbjørn Lien

**Affiliations:** 1 AquaGen AS, P.O. Box 1240, 7462 Trondheim, Norway; 2 Centre for Integrative Genetics, Department of Animal and Aquacultural Sciences, Faculty of Biosciences, Norwegian University of Life Sciences, Ås, Norway; 3 Faculty of Chemistry, Biotechnology and Food Science, Norwegian University of Life Sciences,, Ås, Norway; 4 Faculty of Science and Technology, Norwegian University of Life Sciences, Ås, Norway; 5 Geno Breeding and AI Association, Ås, Norway

## Abstract

**Background:**

Although bovine milk is regarded as healthy and nutritious, its high content of saturated fatty acids (FA) may be harmful to cardiovascular health. Palmitic acid (C16:0) is the predominant saturated FA in milk with adverse health effects that could be countered by substituting it with higher levels of unsaturated FA, such as oleic acid (C18:1*cis*-9). In this work, we performed genome-wide association analyses for milk fatty acids predicted from FTIR spectroscopy data using 1811 Norwegian Red cattle genotyped and imputed to a high-density 777k single nucleotide polymorphism (SNP)-array. In a follow-up analysis, we used imputed whole-genome sequence data to detect genetic variants that are involved in FTIR-predicted levels of C16:0 and C18:1*cis*-9 and explore the transcript profile and protein level of candidate genes.

**Results:**

Genome-wise significant associations were detected for C16:0 on *Bos taurus* (BTA) autosomes 11, 16 and 27, and for C18:1*cis*-9 on BTA5, 13 and 19. Closer examination of a significant locus on BTA11 identified the *PAEP* gene, which encodes the milk protein *β*-lactoglobulin, as a particularly attractive positional candidate gene. At this locus, we discovered a tightly linked cluster of genetic variants in coding and regulatory sequences that have opposing effects on the levels of C16:0 and C18:1*cis*-9. The favourable haplotype, linked to reduced levels of C16:0 and increased levels of C18:1*cis*-9 was also associated with a marked reduction in *PAEP* expression and β-lactoglobulin protein levels. β-lactoglobulin is the most abundant whey protein in milk and lower levels are associated with important dairy production parameters such as improved cheese yield.

**Conclusions:**

The genetic variants detected in this study may be used in breeding to produce milk with an improved FA health-profile and enhanced cheese-making properties.

**Supplementary Information:**

The online version contains supplementary material available at 10.1186/s12711-022-00731-9.

## Background

Bovine milk is a staple food ingredient in billions of people’s diet, where it serves as an important source of proteins, fat, minerals and vitamins. Nonetheless, the positive effects of cow milk on human health have been debated, primarily due to its high content of saturated fatty acids (FA) as compared to the level of unsaturated acids [1, 2]. Palmitic (C16:0) and oleic (C18:1*cis*-9) acids are the dominant saturated and unsaturated milk FA respectively, and together they represent 40 to 50% of the total milk fat content [3]. Replacing dietary saturated with unsaturated fat has been shown to reduce the risk of cardiovascular diseases [1, 4], and might also reduce the risk of insulin resistance and type-2 diabetes [5].

Both C16:0 and C18:1*cis*9 have moderate heritability estimates ranging from 0.1 to 0.3 in the extensively studied Holstein–Friesian breed [6–8]. In Norwegian Red cattle, the heritability estimates are equal to 0.13 and 0.14 for C18:1*cis*9 and C16:0, respectively [9], which raises the possibility of using selective breeding to improve the FA profile of cow’s milk.

Detection of causal polymorphisms and implementation of genome information in selection typically involves the use of phenotypic data from thousands to, even, millions of individuals [10]. Traditionally, characterisation of milk fat composition has been performed using gas chromatography (GC), but this becomes costly when thousands of samples must be analysed. An alternative is to predict milk fat composition using Fourier transform infrared spectroscopy (FTIR) [9, 11–15], which produces fast, cheap and detailed phenotypes.

Compared to the widely used single nucleotide polymorphism (SNP) panels, the use of whole-genome sequence data has the potential to detect causative variants underlying a given trait, or at least genetic variants that are in very close linkage disequilibrium (LD) to the causative variants. Once identified, such variants can be used to develop cost-effective genotyping panels for validating quantitative trait loci (QTL) and for more accurate genomic predictions that persist across diverse genetic backgrounds and multiple generations [16, 17]. Moreover, coordinated international actions to generate genome-wide maps of functional elements for animal genomes will provide valuable knowledge to understand the context within which these variants operate, and might eventually pin down the variants and candidate genes underlying the genetic basis of complex traits [18].

In this study, our aim was to identify and improve the current understanding of genetic variants underlying C16:0 and C18:1*cis*-9 content in bovine milk using a combination of imputed sequence data, mRNA- and protein-expression profiling. Initially, FTIR-predicted phenotypes were combined with array-based SNP genotypes in a genome-wide association study (GWAS) to identify QTL that have an impact on the concentration of the two FA. Next, a candidate gene region was fine-mapped using the imputed sequence variants (SNPs and indels). Finally, gene expression data from mammary epithelial cells and milk protein measurements were used to complement the analysis.

## Methods

### Estimation of bovine milk fat composition from FTIR spectroscopy data

Milk fat composition was estimated from FTIR spectroscopy data as described in Olsen et al. [9] with some adjustments for the number of spectra and animals used. In brief, 224 milk samples obtained from a previous feeding experiment and 659 samples from field sampling were analysed in parallel by FTIR and GC with flame ionisation detector (GC-FID) reference analysis. FTIR spectra (regressors) were subsequently calibrated against GC-FID reference values (regressands) by using powered partial least squares regression. Regressands were presented as percentages of GC-FID FA values to total fat to decrease the correlation between the FA and total fat in milk samples to a minimum value. The calibration model was applied to 4,619,737 infrared spectra from 640,304 cows. In this study, we used the C16:0 and C18:1*cis*-9 traits.

A detailed description of the estimation of heritabilities and daughter yield deviations (DYD) is in Olsen et al. [9]. Briefly, the heritability estimates were obtained from a dataset consisting of 2,209,486 FA profiles from 426,505 cows with a pedigree of 716,753 animals using the DMU software version 6 release 5.1 [19].

DYD for 2434 genotyped artificial insemination (AI) bulls were estimated using the 4,619,737 spectra for the full dataset of 640,304 cows with a pedigree of 999,470 animals as the sire averages of daughters’ predicted FA compositions, which were each corrected for daughter fixed effects, non-genetic random effects and half of the genetic effects of the bulls mates [9]. DYD were then used as pseudo phenotypes in later GWAS.

The concentration of the two FA together with the accuracy of prediction (in the form of cross-validated squared Pearson product-moment correlation coefficients; R^2^CV) and heritabilities of the individual animal predicted FA concentrations, were as reported in Olsen et al. [9]. In brief, the calibration of FTIR spectra against GC-FID reference values was assessed by 20-fold cross-validation, i.e. the calibration data were divided randomly into 20 segments and each of them was used as an independent test set at a time [9]. Mean concentrations were 25.3 and 21.4% of total fat for C16:0 and C18:1*cis*-9, respectively. R^2^CV were equal to 0.77 and 0.94 and heritabilities to 0.16 and 0.14, for C16:0 and C18:1*cis*-9, respectively.

### SNP genotyping and imputation

Details on genotyping, DNA extraction and imputation were previously described by Knutsen et al. [15]. In brief, genotypes of the animals were obtained from the routine genotyping of bulls performed by Geno Breeding and AI Association. The bulls were genotyped on at least one of four different platforms in order to make a genome-wide high-density SNP dataset for the association analyses: the Affymetrix 25K SNP array, a custom Affymetrix 50K SNP array, the Illumina 54K BovineSNP50 BeadChip and the 777K Illumina BovineHD Genotyping BeadChip, combined, and imputed to the 777K density. Imputation was done using the Beagle software version 4.1 [20], with effective population size (Ne) set to 200 and number of phasing iterations set to 20. The remaining parameters were set to default values. Map positions were based on the UMD 3.1 reference assembly [21], as this was the most mature assembly available at the time of analysis, but map positions of the ARS-UCD1.2 assembly were also added to the Additional data using the NCBI Genome Remapping Service (https://www.ncbi.nlm.nih.gov/genome/tools/remap).

For each imputation step, several genotype quality control filters were applied: (1) SNPs with a minor allele frequency lower than 0.01 and a Hardy–Weinberg equilibrium *p*-value less than 1e^−7^ were removed; (2) animals with more than 10% Mendelian errors were excluded from the dataset, and all remaining genotypes with Mendelian errors were set to missing and later imputed; (3) SNPs and animals with a call rate lower than 95% were discarded; and (4) for each step, the imputation quality was tested using fivefold cross-validation. SNPs with a mean discordance rate (calculated as percent incorrectly imputed genotypes per marker averaged over each fold) between true and imputed genotypes above 10% were removed, since these SNPs are likely to be misplaced in the reference genome assembly [22]. SNPs on unplaced scaffolds and sex chromosomes were also discarded from the dataset due to insufficient quality.

In total, 2434 genotyped AI bulls were considered for the initial 777k GWAS. After filtering bulls with less than 20 daughters, the dataset contained 1811 animals with imputed genotypes for the 777K Illumina BovineHD BeadChip. Of the 1811 bulls, 57 had genotypes imputed from the Affymetrix 25K array, 237 were imputed from the custom Affymetrix 50K SNP array, 1113 from the Illumina 54K BeadChip, and 404 were already genotyped on the 777K Illumina BovineHD BeadChip. The resulting dataset consisted of 1811 bulls with trait data in the form of DYD based on 20 or more daughters for the relevant FA and with genotypes for 609,361 SNPs distributed over all 29 autosomes. The average number of daughters per bull was ~ 300 in all steps.

### Whole-genome sequencing, variant calling and sequence imputation

Whole-genome sequencing data were obtained from 153 animals (132 AI bulls and 31 cows) as described in Olsen et al. [23]. All reads were aligned against UMD 3.1 using the BWA MEM algorithm version 0.7.10. Variant calling was done with the FreeBayes tool version 1.0.2 [24]. Missing genotypes in the resulting variant call format (VCF)-file were imputed and phased using Beagle version 4.1 [20]. This phased dataset was used as a reference panel for imputing the 1811 animals from high-density panels to sequence density at selected regions with Beagle using the same imputation parameters as described before, except that the allele miscall rate was set to 0.01. In a final filtering step, variants with a minor allele frequency higher than 0.02 were retained. In addition, the variants with a Beagle’s reported allelic R^2^ (AR^2^) lower than 0.7 were filtered, as this has been shown to be a robust and reliable threshold for filtering imputed sequence variants [25–27].

### Genotyping of cows

The 36 cows used for RNA sequencing were also genotyped on the Illumina BovineSNP50 BeadChip. Blood samples were collected by certified personnel, and DNA extraction and genotyping were performed according to the manufacturer's protocol. Genotypes were quality-checked and imputed to sequence density as previously described.

### Genome-wide association study

This study was initiated by conducting a single marker GWAS for C16:0 and C18:1*cis*-9 concentration with genotypes for 609,361 genome-wide distributed SNPs and phenotypes in the form of DYD from 1811 elite AI bulls, with follow-up analyses of a selected region imputed to sequence level density. The initial GWAS was conducted with the GCTA software [28] for computational feasibility, while the follow-up analyses of selected regions were analysed using the ASReml package version 3.0 [29] to be able to weight the analysis by number of daughters for each DYD and to be able to use genotype dosage data in the model.

A mixed linear model single-marker association analysis was performed with the—mlma-loco option of GCTA [30]. The model fitted to the performance information for each trait and each SNP was:

1 $$\mathbf{D}\mathbf{Y}\mathbf{D}=\mathbf{1}\mu +\mathbf{x}\mathbf{b}+\mathbf{Z}\mathbf{a}+\mathbf{e},$$

where $\mathbf{D}\mathbf{Y}\mathbf{D}$ is the vector of bull performances, $\mathbf{1}$ is a vector of ones, $\mu$ is the mean term, $\mathbf{b}$ is the fixed additive effect of the candidate SNP to be tested for association, $\mathbf{x}$ is the SNP genotype indicator variable coded as 0, 1 or 2, $\mathbf{Z}$ is an incidence matrix relating phenotypes to the corresponding random polygenic effects, $\mathbf{a}$ is a vector of random polygenic effects, estimated using a genomic relationship matrix calculated with all SNPs except those on the chromosome where the candidate SNP is located, and $\mathbf{e}$ is the random residual effect. The $var(\mathbf{a})$ will be re-estimated each time a chromosome is excluded from calculating the genomic relationship matrix. The suggestive significance level was set to *p* = 1e−5, which is the default setting in the R-package qqman [31] used for producing manhattan plots, representing a more lenient significance threshold for a potential follow-up. The genome-wise significance level was set to 8.2e−8, corresponding to a nominal type I error rate of 0.05 and Bonferroni correction for 609,361 SNPs.

### Re-analyses of the candidate gene region on BTA11 using sequence-level variants

All sequence-level polymorphisms located between 90 and 107 Mb on BTA11 that passed quality control (102,021 variants) were analysed for association with C16:0 and C18:1*cis*-9 content using ASReml. The model that was fitted to the information on performance for each trait—marker combination was:

2 $$\mathbf{D}\mathbf{Y}\mathbf{D}=\mathbf{1}\mu +\mathbf{x}\mathbf{b}+\mathbf{Z}\mathbf{a}+\mathbf{e},$$

where $\mathbf{D}\mathbf{Y}\mathbf{D}$ is the vector of bull performances weighted by the number of daughters, $\mathbf{1}$ is a vector of ones, $\mu$ is the overall mean, $\mathbf{x}$ is a vector of marker genotypes coded as a decimal number between 0 and 2 depending on the estimated dosage of the alternate allele (as reported by Beagle 4.1), $\mathbf{b}$ is the fixed effect of the marker, $\mathbf{Z}$ is an incidence matrix relating phenotypes to the corresponding random polygenic effects, $\mathbf{a}$ is a vector of random polygenic effects, and $\mathbf{e}$ is a vector of residual effects. Genetic and residual variances were estimated from the data. $\mathbf{a}$ was assumed to follow a normal distribution ~ $N\left(0,\mathbf{A}{\upsigma }_{\mathrm{A}}^{2}\right)$ where $\mathbf{A}$ is the relationship matrix derived from the pedigree, and ${\upsigma }_{\mathrm{A}}^{2}$ is the additive genetic variance. $\mathbf{e}$ was assumed to follow a normal distribution ~$N\left(0,\mathbf{W}{\upsigma }_{\mathrm{e}}^{2}\right)$ where ${\upsigma }_{\mathrm{e}}^{2}$ is the environmental variance and $\mathbf{W}$ is the matrix of weights computed by ASReml based on the number of daughters in the DYD mean. Association analysis was performed for each marker. Since ASReml does not automatically output p-values for the marker effect, these were calculated from the F statistics for the conditional sum of squares, the numerator degrees of freedom and the denominator degrees of freedom with the R-function pf() from the stats package version 3.4.0 [32].

To estimate the proportion of genetic variance explained by all the top SNPs for each trait, the genotypes of markers with a p-value passing Bonferroni correction was extracted and GCTA reml was run with and without the top SNPs as fixed effects using the qcovar option. The resulting drop in genetic variance will give an indication of the proportion of genetic variance explained by these QTL.

### Haplotype analyses

Pairwise LD measurements (r^2^) were estimated and haplotypes were identified for the top-ranking markers within the QTL region using the Haploview 4.2 software [33] on phased genotypes. Haplotypes were defined by Haploview according to the confidence intervals strategy [34].

### RNA isolation, sequencing and read mapping

Gene expression levels were obtained using read counts from mRNA isolated from somatic milk cells (SMC) of 36 cows from the research herd at the Norwegian University of Life Sciences, Ås, Norway. The cows were in different parities due to the limited size of the research herd. All cows were fed the same diet. The animal pedigree was used to avoid selection of close relatives. All milk samples were collected approximately 50 days (range 47–55) after calving. This sampling period was chosen since it roughly coincides with the peak expression of several relevant genes involved in bovine milk fat synthesis [35] and with the top of the lactation curve of Norwegian Red cows [36].

In our study, we isolated mRNA from SMC. However, most studies use mammary tissue from biopsies, which is an invasive sampling technique that has technical challenges and management issues in the recovery of the animals. In contrast, milk is excreted by the mammary epithelial cells (MEC) lining the inside of the udder, which are subject to turnover and shed into the milk and therefore represent a proportion of the somatic cells found in milk [37]. Cánovas et al. [38] found that, compared to other sources (e.g. mammary gland tissue, laser-dissected MEC), the quality of the total RNA extracted from SMC was high. Moreover, the expression profile of genes investigated in SMC-derived material was highly correlated with the expression observed in laser-dissected MEC. Several studies have confirmed the usefulness of this method [37, 39, 40].

Milk samples were collected manually 2 to 3 h after milking to maximise the number of viable cells present in the milk. Teats were cleaned with water followed by 70% ethanol before milking by hand, and 2 × 50 ml milk samples from each animal were collected in Falcon tubes. Samples were stored on ice immediately after collection and centrifuged at 4 °C for 10 min at 2300*g* within 1.5 h to collect the cells that are at the bottom of the tubes. After centrifugation, most of the fat layer was removed with a clean pipette tip and the supernatant was decanted. Each pellet was dissolved in 4 mL 1 × PBS by pipetting up and down and the liquid was transferred to a new 50 mL Falcon tube. Samples were centrifuged at 4 °C for 10 min at 2300*g* and the supernatant was decanted. Cell pellets were dissolved in 1 mL Trizol (Qiagen), and cells were lysed by pipetting up and down. Samples were stored at − 80 °C until RNA extraction with Qiagen RNeasy Plus Universal Tissue Mini Kit (Qiagen) according to the manufacturer’s protocol. RNA concentrations and quality were measured with a NanoDrop8000 spectrophotometer (Thermo Fisher Scientific) and Agilent RNA 6000 assay on Agilent BioAnalyzer 2100 (Agilent Technologies), respectively. All samples had an RNA integrity number (RIN) between 6.6 and 9.2. Samples were prepared for paired-end sequencing (2 × 150 bp) using the Illumina® TruSeq® stranded mRNA library preparation kits and sequenced by the Norwegian Sequencing Centre (www.sequencing.uio.no) using the Illumina HiSeq 3000 platform.

Before mapping, raw read quality was assessed using fastQC version 0.11.5 https://www.bioinformatics.babraham.ac.uk/projects/fastqc/), Illumina adaptors were removed, and the sequences were quality-trimmed using cutadapt [41]. Cutadapt was set to cut adaptors with a minimum overlap length of 8 and low-quality 3′ ends were removed by setting a quality threshold of 20 (phred quality + 33). An index of the UMD 3.1 reference genome was built, and reads were aligned to the reference genome using STAR version 2.3.1 [42]. Sorting and indexing of the resulting BAM files were completed using SAMtools version 1.3 [43]. The code for the described RNAseq mapping method is available as part of a bash-script pipeline found at https://gitlab.com/fabian.grammes/RNAseq-analysis/ (version 1.1.0). To look for novel splice variants of candidate genes, the BAM-files were assembled into transcripts using stringtie version 1.3.3 [44]. Isoform fraction was set to 5%. All other settings were set to default values.

### Effect of genotype on gene expression

A weakness that we identified in the use of somatic milk cells as the basis for RNAseq analysis was that the expression levels of FA metabolism genes varied considerably between the sampled cows. Even after accounting for sequence library size, there was an approximately 100-fold difference in the expression level of key FA metabolism genes (such as *FABP3*, *SCD1* and *DGAT1*) between samples with the highest and lowest levels of expression. Given that we collected the samples from cows raised on the same diet and kept in the same environment at the same lactation stage, we believe that the variation in FA metabolism gene expression level was due to variation in the proportion of MEC compared to white blood cells (immune cells) in each sample. To adjust for this, we included an effect of the total expression level of the other five major milk protein genes (*CSN1S1*, *CSN2*, *CSN1S2*, *CSN3* and *LALBA*) as a covariate in the linear model run by Matrix eQTL [45]. The use of this covariate will be an indirect way of adjusting for the sample MEC to white blood cell fraction.

The percentage of *PAEP* expression variance explained by the top-SNP genotype was calculated by modelling the expression as a function of the animal genotype using the lm function in R.

### Allele-specific expression

Allele-specific expression (ASE) analysis was done using the ASEReadCounter tool from the Genome Analysis Toolkit [46] with default settings. Before running the tool, duplicated reads were removed using markdup from Sambamba [47]. ASEReadcounter produces a table with separate read counts for each heterozygous bi-allelic variant in the provided BAM files. To test for significant levels of ASE, we used a two-sided exact binomial test with the R-function binom.test and with the number of trials equal to total read counts at each locus. The test gives a *p*-value for the hypothesis that the number of reads for each allele at heterozygous loci will be approximately equal when sequenced [48]. The *p*-values were adjusted using the p.adjust R-function with method = “bonferroni”.

### Protein analysis

The relative concentration of β-lactoglobulin (β-LG) was determined by using an Agilent capillary electrophoresis (CE) system (G1600AX), installed with the Agilent ChemStation software (Agilent Technologies, Germany) as described in Ketto et al. [49]. The concentration in β-LG was determined by adjusting the relative concentration of β-LG with the total protein content determined by MilkoScan FT1 (Foss Electric A/S, Hillerød, Denmark). The effects of milk protein genotypes on the concentration of β-LG in milk were analysed using the lme4 R package [50], where the effect of cow was treated as a random effect. Effects of parity, selection line and stage of lactation were not significant and therefore they were excluded from the statistical analysis.

### Variant annotation

All variants were annotated using the Ensembl Variant Effect Predictor web tool [51], based on the Ensembl *Bos taurus* annotation release 88 (ftp://ftp.ensembl.org/pub/release-88/).

## Results

### Genome-wide association analyses on a high-density SNP dataset

**Fig. 1:**
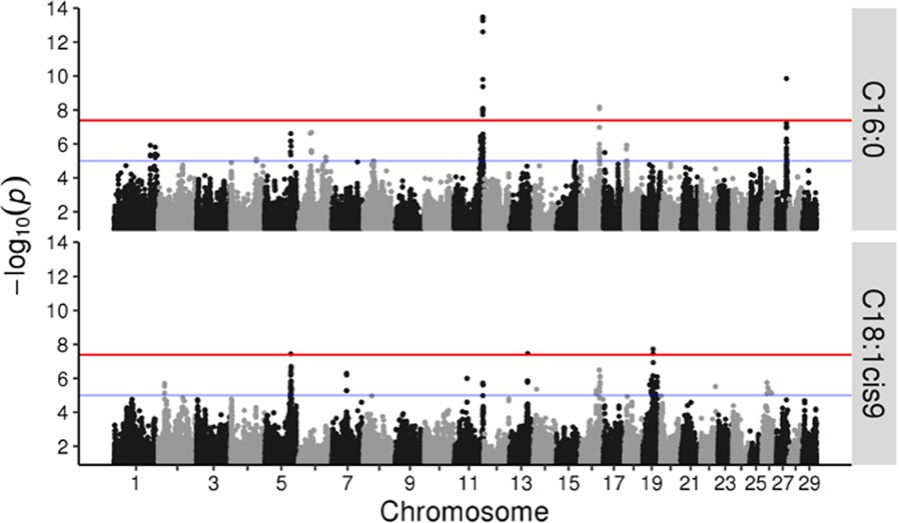
Manhattan plots of GWAS results for C16:0 (top) and C18:1*cis*-9 (bottom). Chromosomes and marker order are represented on the x-axis, with the significance of association (−log_10_ *p*-value) between each marker and trait shown on the y-axis. The red line represents the genome-wise significance level (*p*-value < 4.1e−8), while the blue line represents the suggestive significance level (*p*-value < 1e−5)

To identify chromosomal regions with a major impact on C16:0 and C18:1*cis*-9 levels, we performed an initial GWAS using 1811 animals genotyped for 609,391 SNPs*.* As shown in Fig. 1, genome-wise significant associations (*p*-value < 4.1e−8) were detected for C16:0 level on BTA11, 16 and 27, and for C18:1*cis*-9 level on BTA5, 13 and 19. Suggestive findings (*p* < 1e−5) were detected on BTA1, 4, 5, 6, 8, 17 and 18 for C16:0 level and on BTA2, 7, 11, 14, 16, 22 and 26 for C18:1*cis*-9 level (Fig. 1). Results for all significant marker and trait combinations are in Table S1 (see Additional file 1: Table S1).

The most significant associations were between C16:0 level and five SNPs spanning a 24-kb region located at 103.3 Mb on BTA11. This region included the *progestagen-associated endometrial protein* (*PAEP*) gene encoding β-lactoglobulin (β-LG) and the *glycosyltransferase 6 domain containing 1* (*GLT6D1)* gene encoding a protein of the same name. The two top SNPs for C16:0 level had equal p-values and frequencies (*p*-value = 3.34e−14, MAF = 0.34). The first (rs110186753; *A*/*G*) is situated in intron 1 of the *PAEP* gene at 103,302,351 bp, and the second rs109087963 (*G*/*A*) is located 1940 kb downstream of *PAEP* at 103,308,330 bp. These SNPs also showed an association with C18:1*cis*-9 level (*p*-value 1.91e−6), with alleles having opposing effects. That is, the *G* and *A* alleles of rs110186753 and rs109087963, respectively, were associated with elevated levels of C16:0 and reduced levels of C18:1*cis*-9. The proportion of genetic variance explained (2p(1-p)α^2^/σ^2^A) by each of these SNPs was 3.4% for C16:0 level (allele substitution effect: 0.18 g/100 g milk fat) and 1.4% for C18:1*cis*-9 level (allele substitution effect: − 0.12 g/100 g milk fat).

### Fine-mapping of the QTL region on BTA11

**Fig. 2:**
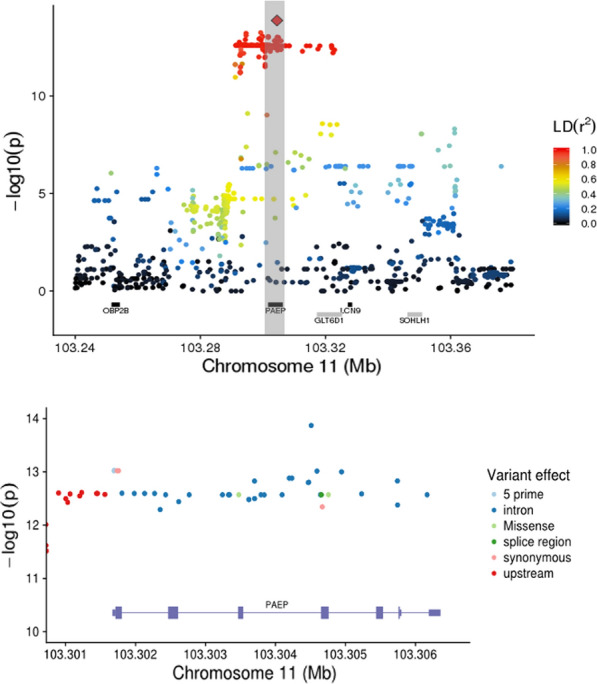
Analysis of C16:0 using sequencing data. (Top) Association analysis of C16:0 in the region between 103.2 and 103.4 Mb on BTA11 using variants imputed from sequence data. The zoomed region showed in the bottom figure, is indicated with a vertical grey bar. The y-axis shows −log_10_(*p*-value) for each marker-trait association, while the x-axis denotes marker position. The red diamond indicates the most significant marker for C16:0; rs110992345 at 103,304,509 bp. Colouring indicates the level of LD (r^2^) between each marker and rs110992345. Gene annotation information according to Ensembl annotation release 88 is shown with grey and black bars reflecting positive and negative strand orientations, respectively. (Bottom) An expanded plot showing variants and their effect relative to the position in the *PAEP* gene. The y-axis shows −log_10_(*p*-value) for each marker–trait association, while the x-axis denotes marker position. Point colour indicates variant effect class according to the Ensembl annotation release 88

To fine-map the QTL on BTA11 and possibly identify underlying causative variants, we re-analysed phenotype data for C16:0 and C18:1*cis*-9 using 109,401 imputed sequence variants spanning a region from 90 to 107 Mb. The results revealed a cluster of 174 variants associated with both C16:0 and C18:1*cis*-9 levels with largely similar *p*-values, MAF and allele substitution effects (Fig. 2). Alleles associated with increased concentration of C18:1*cis*-9 were linked to reduced concentrations of C16:0 and vice versa. The proportion of genetic variance explained by the QTL passing Bonferroni correction for each trait was 0.11 for C16 (QTL on BTA11, 16, and 27) and 0.15 for C18:1 (QTL on BTA5,11,13, and 19). Results for all significant marker and trait combinations are in Table S2 (see Additional file 2: Table S2).

Closer examination of pairwise linkage disequilibrium (LD) measurements (r^2^) between variants in the region, revealed that all 174 variants were in almost perfect LD with each other and could be combined into two major haplotypes extending from ≈10.5 kb upstream of the *PAEP* transcription start site, through the *PAEP* gene and into the neighbouring gene *GLT6D1* (Fig. 2). Two predominant haplotypes had frequencies of 0.29 and 0.54, while less frequent haplotypes, differing from the two major haplotypes only by two and three SNPs, were found with frequencies of 0.04 and 0.06. Two missense variants (rs110066229 in exon 3 and rs109625649 in exon 4) encode the A and B variants of the protein *β-LG* encoded by *PAEP* [52], and were present in the identified haplotype block. Accordingly, our two major haplotypes were denoted A and B. The more frequent B haplotype includes alleles associated with reduced levels of C16:0 (allele substitution effect: − 0.2 g/100 g milk fat) and increased levels of C18:1*cis*-9 (allele substitution effect: 0.14 g/100 g milk fat), i.e. the desirable FA ratio. Table S3 (see Additional file 3: Table S3) provides a more detailed description of the 174 markers assembling the haplotype block, including the haplotype A and B alleles and variant effect predictions.

The haplotype included variants in both the coding and regulatory regions of *PAEP*. After variant annotation, a polymorphism in exon 3 (rs109990218 at 103,304,656 bp) was found to potentially affect alternative splicing of exons in different transcripts (Fig. 2), but no transcript splice variants (freq. > 0.05) were found. The most significant SNP for C16:0 level was situated in intron 3 of *PAEP* (rs110992345; 103,304,509 bp, *p* = 1.35e−14), while the top-ranking marker for C18:1*cis*-9 level was 2 kb upstream of *PAEP* (rs110920335; 103,300,718 bp, *p*-value = 1.35e−8), but no obvious causal function could be assigned to either of these SNPs. Tightly linked to these top SNPs, and highly significant, were the two known missense variants determining the β-LG A and B variants. Lastly, the haplotype block contained two variants in the 5’ untranslated region of *PAEP*, a region that might influence gene expression (rs41255685 at 103,301,690 bp and rs41255686 at 103,301,694 bp both with a *p*-value of 9.5e−14).

### Gene expression analyses

To investigate whether any of the significant variants within the two haplotypes were associated with differential gene expression of the two genes spanned by the haplotype block (i.e. whether they generate a cis expression QTL effect; cis eQTL), mRNA was isolated from somatic milk cells and sequenced to quantify expression of the genes. Although it is included in the QTL region, *GLT6D1* was not found to be expressed in any sample. In contrast, *PAEP* was highly expressed in all samples. Therefore, subsequent analyses were directed towards this gene.

**Fig. 3:**
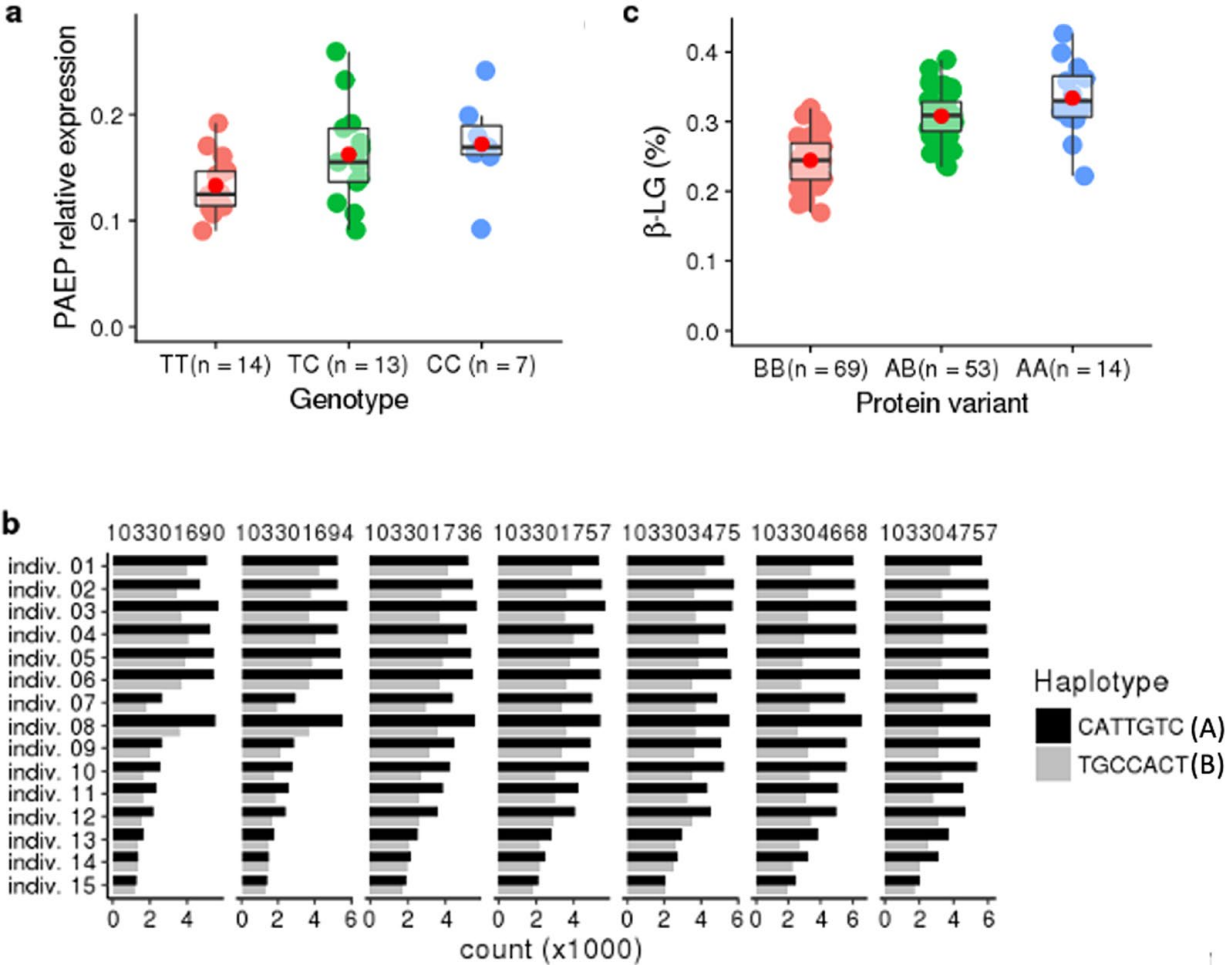
Effects of the top associated variants on expression of the *PAEP* locus. **a** Relationship between cow genotypes (n = 34) of the top associated variant (Chr11_103304509_*T*_*C;* rs110992345) and expression of *PAEP.* The Y-axis denotes the expression of *PAEP* relative to the sum of expression of the five other milk protein genes. The red dot represents the mean expression value within each group. **b** ASE for 15 cows heterozygous for seven exonic SNPs (position shown in bp on BTA11) within the *PAEP* gene. The X-axis shows mean normalised counts (× 1000) per haplotype allele. Haplotype A is coloured black, and haplotype B is coloured grey. **c** The relationship between the two β-LG protein variants and the percentage of β-LG measured in 136 milk samples. The red dot represents the mean expression value within each group

SNPs that were significant at the genome-wide level, and/or situated within a region extending 5 kb up- and downstream from *PAEP*, were tested for their association to the expression level of *PAEP* adjusted by total read count of all measurable milk protein mRNAs (see “Methods” Section). The analysis showed that all 93 tested polymorphisms were significantly (*p*-value < 0.03) associated with *PAEP* expression (see Additional file 4: Table S4). Their association (*p*-values) was relatively similar, reflecting the similarity in allele frequency and LD between the tested variants. To illustrate this, the *PAEP* expression levels relative to genotypes for rs110992345, which is the marker most significantly associated with C16:0 level, is shown in Fig. 3a. In Fig. 3a, the *T* allele of rs110992345 which is present in the frequent and favourable B haplotype, and hence associated with lower *PAEP* expression, is compared to the *C* allele found in the A haplotype.

To validate the apparent difference in allele-dependent expression levels, we also tested for ASE in the 15 animals that were heterozygous for the seven variants located in exons and untranslated regions (UTR) of *PAEP*. Concordant with the results of the eQTL analysis, we found that in 98 out of 105 tests for ASE, the alleles present in the B haplotype were expressed at a significantly (adjusted *p*-value < 0.05) lower level than the alleles present in the A haplotype (Fig. 3b). Fifty of the ASE-tests showed extremely low adjusted *p*-values (< 5.3e−50), with the most significant having 6598 reads from the A haplotype and 2635 reads from the B haplotype (see Additional file 5: Table S5).

### Protein analyses

Finally, β-LG protein levels were quantified to test whether the haplotypes associated with differences in FA and *PAEP* expression levels also reflect differences in protein concentration level. One-hundred and thirty-six cows were genotyped for the two SNPs determining the A and B *β-LG* variants tagging the A and B haplotypes, respectively. The results showed that animals homozygous for the B variant of *β-LG* (i.e. alleles of haplotype B) had on average 35% less β-LG than cows homozygous for the haplotype tagged by the A variant (Fig. 3c).

## Discussion

C16:0 and C18:1*cis*-9 are the most abundant FA in bovine milk, but may have opposite effects on human health [1, 4, 5], and genome-based selection strategies increasing the ratio of C18:1*cis*-9 to C16:0 in milk may offer ways to improve fat composition. In the current study, we combined milk FA composition phenotypes with high-density SNP information and whole-genome sequence data, followed by gene expression and protein level analyses to detect genetic variants that influence the levels of these two FA in milk from Norwegian Red cattle.

The results revealed genome-wise significant QTL for C16:0 level on BTA11, 16 and 27, and for C18:1*cis*-9 level on BTA5, 13 and 19. Subsequent analyses focused on the QTL on BTA11 since it was the most significant and showed opposite effects on levels of C16:0 and C18:1*cis*-9. This analysis revealed a haplotype block spanning multiple variants in regulatory and coding regions of the *PAEP* gene, including the two SNPs coding for the A and B variants of the *PAEP* gene product β-LG. The most frequent haplotype in the block (haplotype B, encoding the B protein variant) was associated with (i) a more favourable C16:0 to C18:1*cis*-9 ratio, (ii) lower *PAEP* expression and (iii) lower β-LG levels as compared to haplotype A.

Although this study detected variants in the *PAEP* gene that have an effect on milk FA, several previously published GWAS have not identified variants near *PAEP* that significantly affect milk FA composition [53–56]. In this work, only predicted FA profiles with solid prediction accuracies were used (0.77 for C16:0 and 0.94 for C18:1cis-9). Still, we cannot rule out the possibility that absorption patterns from other molecules in milk or other correlated FA created a false positive signal. The current study was conducted using Norwegian red cattle, that may carry causative variants segregating private to this breed, which may explain why SNPs in the *PAEP* gene were found to be significant here, and not in most other studies. In a study using Holstein, Jersey, and crossbred cows and MIR predicted FA profiles in Australia, SNPs in *PAEP* were indeed found to significantly affect C18:1 concentration [57].

β-LG is the most abundant whey protein in bovine milk [58]. The two major protein isoforms, variants A and B, differ at mRNA positions 64 and 118 leading to ASP > GLY and VAL > ALA substitutions, respectively [52]. The association between *PAEP* allelic variants and milk production traits such as protein percentage, total fat yield and fat percentage in cows has been well documented [59, 60]. Previous studies have shown that β-LG can bind both saturated and unsaturated FA, especially C16:0, in vitro [61]. In dairy sheep, β-LG variants were shown to affect the concentration of C16:0 along with other FA [62]. Furthermore, the B variant associated with reduced C16:0 levels has been linked to favourable chemical composition and technological parameters such as shorter coagulation time, a lower concentration of whey proteins together with higher casein levels and higher cheese yield [49, 63].

Still, the mechanism that underlies how different β-LG variants or the β-LG protein concentration in milk could influence individual FA is not understood. However, given the strong C16:0 binding capacity of β-LG, the QTL effect on the C16:0 to C18:1*cis*-9 ratio may be caused by differences in the affinity for the FA between the protein variants, a change in the concentration of β-LG due to differential expression of *PAEP*, or a combination of these effects.

Although differential expression of the two protein variants was evidenced, we consider that this difference is more likely related to linked polymorphisms within regulatory regions rather than within the protein variants themselves [64, 65]. *PAEP* expression in lactating mammals is reported to be regulated by signal transducer and activator of transcription 5 (STAT5, also known as milk protein binding factor) and activator proteins 1 and 2 [66]. Several polymorphisms located in putative binding sites for these transcription factors have been identified [67–69], but the extensive levels of LD in the region hamper our ability to pinpoint one specific variant as the underlying causative factor. However, several of our top-ranked variants were located in these binding sites. Thus, we hypothesize that the effect on gene expression can be due to the combined impact of alterations at several regulatory sites within the haplotypes, rather than to one specific SNP.

In addition to the *PAEP* gene, our GWAS highlights several other genes with functions related to milk FA composition. For example, the QTL on BTA5 at 93.9 Mb affected both C16:0 and C18:1*cis*-9 levels in opposite directions, with the most significant SNP for C18:1*cis*-9 level being situated in the first intron of the *microsomal glutathione S-transferase 1* (*MGST1*) gene. Although the role of this gene in milk fat synthesis is unclear, it is known to be strongly associated with levels of milk fat, protein, and milk yield [25, 70, 71].

BTA13 harbours a QTL for C18:1*cis*-9 level in a region that also affects de novo-synthesis of short- and medium-chained saturated acids (especially C8:0) in our population [9, 15]. This QTL region contains at least two functional candidate genes, *nuclear receptor coactivator 6* (*NCOA6*) at 64.6 Mb and *acyl-CoA synthetase short-chain family member 2* (*ACSS2*) gene at 64.8 Mb. ACSS2 facilitates the conversion of acetate to acetyl-CoA early in the de novo synthesis of FA [35], while NCOA6 is a transcriptional coactivator enhancing, among other things, the activity of the *peroxisome proliferator-activated receptor gamma* (*PPARG)* gene, which encodes a well-described transcriptional regulator affecting lipid storage [35, 72, 73].

Two distinct QTL were found for C18:1*cis*-9 level on BTA19, among which that at 51.38 Mb was located near the *fatty acid synthase* (*FASN*) gene, which encodes a multifunctional enzyme that catalyses de novo synthesis of milk FA [35].

We also detected chromosome-wise significant associations between C18:1*cis*-9 level and markers situated near the *stearoyl-coenzyme A desaturase 1* (*SCD*) gene on BTA26. SCD is involved in the synthesis of monounsaturated FA by introducing a double bond in the delta-9 position of C14:0, C16:0 and C18:0, primarily, thus producing the *cis*-9 variant of these acids [74].

The QTL affecting C18:1*cis*-9 level at 36.2 Mb on BTA27 spans the *glycerol-3-phosphate acyltransferase 4* (*GPAT4*) gene, which encodes the rate-limiting enzyme in the triacylglycerol biosynthesis pathway and plays a crucial role in milk fat biosynthesis [75].

The single gene with the most pronounced effect on milk fat composition reported in several other breeds, is *diacylglycerol O-acyltransferase 1* (*DGAT1*) [56, 76]. The reported variants were not found to segregate in the whole-genome sequence data of our bulls, and we believe it is likely that this polymorphism has reached fixation in Norwegian Red.

As discussed in the previous paragraphs, QTL for C16:0 and C18:1 levels were found in several regions of the genome. The current study extends our previous work reported in Olsen et al.[9]. Although the QTL detected in our previous study [15] that investigated de-novo synthesized FA largely overlapped with QTL reported in Olsen et al. [9], those for C16:0 and C18:1 levels in Olsen et al.[9], were not confirmed in the work described in this paper. We believe that the main reason for these discrepancies is that our data material, especially the marker density, have been increased markedly compared to that used in Olsen et al. [9]. While the GWAS of Olsen et al. [9] included only 17,000 SNPs, the current GWAS incorporated more than 600,000 markers imputed from 50 to 777k density. In addition, the current work included DYD estimates using spectra from a much larger number of cows compared to the previous work, leading to variability in the DYD values between the two data sets. Notably, the number of genotyped bulls with DYD for the analysis was also doubled, from ~ 900 to ~ 1800 bulls.

An essential requirement when using phenotype data (FA composition) from FTIR profiles is that individual acids are predicted with high confidence. The prediction accuracy of mid-infrared spectroscopy has been demonstrated [9, 11, 13, 14, 77–80]. However, since FA are correlated to total fat, a possible concern is that the predicted values reflect total fat rather than individual acids [81]. To address this, we assessed FA concentrations as percentages of total fat instead of gram-acid-per-unit-of-milk [9], which led to a prediction accuracy (in the form of cross-validated squared Pearson product-moment correlation coefficients) of 0.77 for C16:0 and 0.94 for C18:1*cis*-9 levels. Soyeurt et al.[77] suggested that the predicted concentrations were due to real absorbance values specific to the FA if the calibration correlations were higher than the correlations between total fat and FA. As reported in Olsen et al. [9], the C16:0 and C18:1*cis*-9 squared correlation to total fat was 0.19 and 0.03, respectively, which is markedly lower than the cross-validated squared Pearson product-moment correlation coefficients. A consequence of correcting for total fat is that the prediction accuracies are expected to be lower than when FA concentrations are expressed as a quantity per unit of milk [13, 77, 78]. This was the case for C16:0, while the prediction accuracy of C18:1*cis*-9 was found to be comparable to those obtained by milk-based models [9, 13, 78].

Previous work from our group and others has shown that the concentrations of milk FA can have strong genetic and phenotypic correlation to each other [11, 82, 83]. C16:0 and C18:1 levels have been found to be negatively correlated to each other, but less to other FA [11]. Thus, selection for both traits in a desired direction could be feasible. Also, the prediction of one of these FA could depend on the prediction of the other. In addition, if this correlation holds also for future samples in other environments, this correlation could be used to achieve reliable predictions [11].

In recent years, methods that explore ways to apply imputed sequence variants in GWAS and genomic predictions in dairy cattle have emerged [17, 84, 85]. The current study used sequence imputation to fine map a QTL region associated with 16:0 and C18:1*cis*9 levels in milk. With sequence density genotypes, we expect the causative variants to be present in the data for the direct estimation of their GWA *p*-value, and hence also their effect on the trait. While GWAS with imputed sequence data have previously confirmed causative loci in cattle [86], imperfect imputation, extensive LD and sampling error may result in the causative polymorphism not being identified as the most highly associated variant. However, using non-linear prediction models where most variant effects are set to zero and some are set to moderate or large values, seems promising [22, 86]. Other studies have shown improved genomic prediction reliabilities when including selected sequence variants from GWA in the prediction [17, 85]. Both these strategies could be used with our results. Nonetheless, further research to discover functional variants in the genome, and improvements to the computational and statistical methodology of GWA and genomic prediction strategies is critical to realising the full potential of the sequence data approach.

## Conclusions

The current study revealed a haplotype block with two major haplotypes spanning both coding and regulatory sequences of the *PAEP* gene, including the polymorphisms underlying the A and B variants of the β-LG protein. The most frequent haplotype B was associated with an altered C16:0 to C18:1*cis*-9 ratio and a marked reduction in *PAEP* expression and β-LG levels, which suggests a regulatory role of the causative variants that underlie the QTL. Furthermore, the B variant is considered to be beneficial for technological cheese production traits. Thus, our results may be applied in breeding to produce milk with a potentially healthier FA profile and more favourable cheese-making properties.

## Supplementary Information

**Additional file 1: Table S1**. GWAS results for C16:0 and C18:1*cis*-9 levels. All significant (*p* < 1e−5) marker—trait combinations from the GWA analysis.

**Additional file 2: Table S2**. Results for single-marker association analyses (*p* < 1e−5) for C16:0 and C18:1*cis*-9 levels on imputed sequence data in the region between 100 and 107 Mb on BTA11.

**Additional file 3: Table S3**. Detailed information of the 174 markers included in the haplotype block with opposite effects on C16:0 and C18:1*cis*-9 levels, with haplotype alleles and variant effect predictions from Ensembl.

**Additional file 4: Table S4**. Results from the eQTL analyses showing 93 significant variants with the p-values for the GWAS and eQTL linear model.

**Additional file 5: Table S5**. Results from the 105 binomial tests for ASE (statistical significance of deviations from the theoretically expected distribution of reads originating from the two alleles of a heterozygous SNP) conducted on 15 animals that were heterozygous for the seven variants located in exons and UTRs of the *PAEP* gene*.*

## Data Availability

The datasets supporting the conclusions of this article are included within the article and its additional files. DNA and RNA sequence data will be submitted to the European Nucleotide Archive, http://www.ebi.ac.uk/ena. Phenotype and genotype data are available only upon agreement with Geno Breeding and AI Organization (http://www.geno.no).
